# Unlocking the Neuroprotective Potential of *Semecarpus anacardium* L.—An Updated Review

**DOI:** 10.3390/antiox15060660

**Published:** 2026-05-24

**Authors:** Sureshbabu Ram Kumar Pandian, Subramanian Haripriya, Renganathan Seenivasagan, Tong Woei Yenn

**Affiliations:** 1 Department of Biotechnology, Kalasalingam Academy of Research and Education, Krishnankoil 626126, Tamil Nadu, India; s.haripriya@klu.ac.in; 2 Institute of Medical Science Technology (UniKL MESTECH), Universiti Kuala Lumpur, A1-1, Jalan TKS 1, Taman Kajang Sentral, Kajang 43000, Selangor, Malaysia; wytong@unikl.edu.my; 3 Department of Biotechnology, Arulmigu Kalasalingam College of Arts and Science, Krishnankoil 626126, Tamil Nadu, India; r.seenivasagan@akcas.in

**Keywords:** *S. anacardium*, crude extract, oxidative stress, inflammation, neurodegeneration, health and well-being

## Abstract

Neurodegenerative diseases (NDs) pose a significant health burden globally, and this burden is increasing with an ageing population. Despite this challenge, restorative treatments for NDs remain elusive. In these conditions, the brain is vulnerable to oxidative stress and inflammation due to a deficiency or reduction in antioxidative enzymes. Oxidative stress and inflammation damage neuronal cells, leading to neurodegeneration. Various studies have explored the neuroprotective effects of flavonoids in different in vitro and animal models, primarily due to their antioxidative and anti-inflammatory properties. Crude extracts and active metabolites of *Semecarpus anacardium* L. have shown potential in reversing dysregulated oxidative stress and neuroinflammation. *S. anacardium* L. extract (SAE) and its phytocomponents, such as butein, anacardic acid, and amentoflavone, have been experimentally demonstrated to modulate oxidative stress and neuroinflammation through coordinated activation of Nrf2-mediated antioxidant pathways and suppression of NF-ĸB-driven inflammatory signaling. At a molecular level, flavonoids from SAE induce the expression of p38 MAPK and Nrf2, as well as antioxidant enzymes. Furthermore, inflammatory genes such as NF-ĸB, MAPK, AP-1, iNOS, and COX-2 are suppressed following treatment with SAE. NF-ĸB inhibition leads to neuroprotection via inhibiting the function of caspase-3 and apoptosis. Overall, this review discusses the protective role of SAE and its phytocomponents in mitigating neuronal oxidative stress, inflammation, and degeneration. Furthermore, this review highlights the translational potential of SAE and its phytocomponents as complementary therapeutic candidates for neurodegenerative disorders. However, variability in extract composition and limited pharmacokinetic characterization remain key barriers to clinical translation.

## 1. Introduction

### 1.1. Overview of Neurodegenerative Diseases

Neurodegenerative diseases (NDs) are a diverse group of CNS disorders that affect memory, reasoning, emotions, and motor skills [1,2]. The pathology of NDs associated with the dysfunction of the central and peripheral nervous systems and death involves autophagosomal or lysosomal systems, oxidative stress, inflammation, protein misfolding and homeostasis, programmed cell death, genetics, and environmental factors [3]. Common NDs include stroke, brain trauma, prion disease, SCI, ALS, AD, PD, HD, MND, SCA, TBI, and SMA.

### 1.2. Role of Oxidative Stress and Inflammation in Neurodegeneration

**Figure 1:**
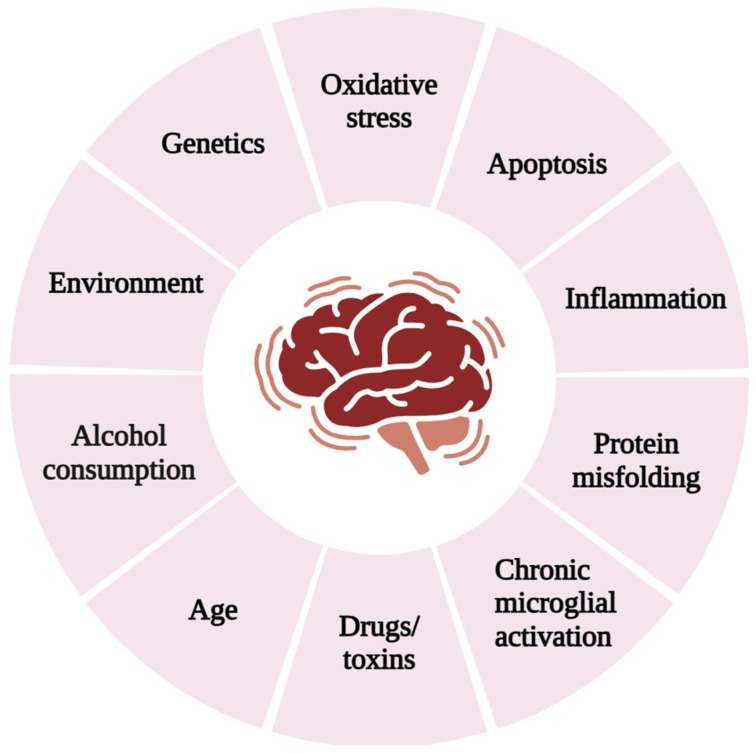
Risk factors contributing to neurodegenerative diseases.

Oxidative damage is the main pathological factor associated with the age-related cognitive changes [4,5]. The brain is particularly susceptible to oxidative stress due to low levels of GSH [6], high polyunsaturated fatty acid (PUFA) content [7], elevated oxygen requirements [8], and a limited antioxidant defense system [9]. Elevated levels of calcium, lipoxygenase, lipid peroxidation, and COX were observed in brain hypoxia, which alters the intracellular microenvironment [10]. Excessive cellular ROS causes oxidative stress, which can activate or repress NF-ĸB signalling depending on the phase and context. NF-ĸB can have both antioxidant and pro-oxidant roles [11] and serves as a regulator of the inflammatory response [12]. Oxidative stress and inflammation contribute cognitively to various disease conditions [13], including neuronal degeneration [14,15]. Various factors inducing neurodegeneration are depicted in Figure 1.

### 1.3. Semecarpus anacardium L.: Ethnopharmacological Background and Relevance

**Figure 2:**
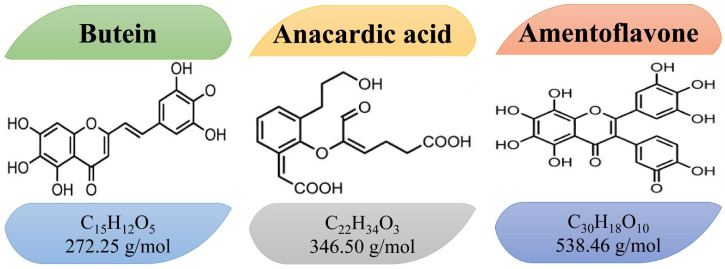
The structure, molecular weight and formula of the phytocompounds butein, anacardic acid, and amentoflavone.

*S. anacardium* L. commonly known as ‘*Bhallataka*’ or ‘*marking nut*,’ is an important medicinal plant classified as ‘*Upavisha*’ (toxic but not lethal for human health) in Ayurveda. Invariably, the *S. anacardium* L. nut contains a wide range of biflavonoids and bioflavonoids, and its extracts have been traditionally used for their neuroprotective effects. *S. anacardium* L. is classified as a Schedule (1) drug, and purification must be performed prior to administration [16]. The presence of phytochemical constituents in *S. anacardium* L. depends on the solvent used for extraction. Acetone extraction yielded the highest amounts of phytochemicals, with the following values (mg/g): total phenols 162.56, flavonoids 82.62, saponins 109.09, and tannins 42.25 [17]. Butein (3,4,2′,4′-tetrahydroxychalcone) [18,19], anacardic acid [16], and amentoflavone [20] are the principal phytocompounds of *S. anacardium* L. The presence of anacardic acid was 5.62% after extraction using the Siddha purification process [16]. HPTLC quantification reported a total amentoflavone content of 10 g/kg in *S. anacardium* seed. However, no report is available for butein. The structure, molecular weight, and formula are represented in Figure 2. They have been extensively studied for their roles in oxidative stress, neuroinflammation, and neuroprotection in both in vitro and in vivo settings. The botanical characteristics, pharmacological applications [21] and toxicological effects [22] of *S. anacardium* L. have been reported earlier. This review discusses the role of *S. anacardium* L., and its selective phytocompounds in protecting against neuronal oxidative stress, inflammation, and degeneration.

## 2. Oxidative Stress and Inflammation Mediated Neuronal Damage

The brain heavily relies on oxidative metabolism; therefore, oxidative stress is regarded as a key factor in neurodegeneration. As shown in Figure 1, several factors such as smoking, radiation, alcohol intake, heavy metals, processed food additives, and others induce oxidative stress in neuron cells. The antioxidant enzymes involved in neuronal degeneration are discussed here.

### 2.1. Glutathione Peroxidase (GPx)

Glutathione peroxidase (EC 1.11.1.9 and EC 1.11.1.12) is a common name for a family of several isozymes that catalyze the reduction of H_2_O_2_ or organic hydroperoxides to water or alcohols through reduced glutathione [23]. GPx enzymes are selenium-dependent and classified as follows: (1) GPx1, found in the cytosol, nucleus, and mitochondria; (2) GPx2, which accumulates in the cytosol and nucleus; (3) GPx3, present in the cytosol; and (4) phospholipid GPx, located in the nucleus, cytosol, mitochondria, bound to membranes [24,25]. GPx is a key component of the neuronal antioxidant defense network, functioning in coordination with GSH, GST, SOD, and catalase to maintain redox homeostasis [26], neutralizes free radical compounds [27], protects cells from oxidative stress. GPx4, a selenoprotein, is a crucial regulator of oxytosis and ferroptosis. Several studies indicate that reduced levels of GPx lead to neurodegenerative disorders [28,29].

### 2.2. Glutathione (GSH)

GSH is a tripeptide that encompasses cysteine, glutamate, and glycine [30]. GSH is primarily secreted in the liver via de novo and scavenger pathways [31]. GSH redox status plays a significant role in regulating most cellular metabolic processes and maintaining the balance between oxidation and reduction [32,33]. GSH performs an endogenous role in conserving the intracellular antioxidant system, redox equilibrium, cell signalling, gene expression, and cell differentiation [34]. To eliminate ROS or RNS, two GSH molecules are oxidized to produce GSSG, which can then be reduced back into two GSH molecules via GSH reductase to sustain redox homeostasis [35]. During oxidative stress, the oxidized form of GSH inside the cell becomes depleted. Indeed, a decrease in the cellular GSH concentration and an increase in GSSG are considered early stages of apoptotic events [36].

### 2.3. Glutathione S-Transferase (GST)

Multiple forms of GST (EC 2.5.1.18) appear to be an evolutionary response by cells to chemical toxicity and oxidative stress. GSTs are found in various cellular organelles such as the cytosol, mitochondria, endoplasmic reticulum, nucleus, and plasma membrane [37]. GSTS are associated with phase-II detoxification enzymes that protect cellular molecules from reactive electrophiles and facilitate the conjugation of glutathione to a wide range of endogenous and exogenous electrophilic compounds. GSTs are classified into membrane-bound microsomal and cytosolic family members [38]. They play a role in maintaining GSH levels in different cellular compartments and also detoxify endogenous toxic metabolites, superoxide radicals, and exogenous toxic chemicals [39].

### 2.4. Superoxide Dismutase (SOD)

SOD is a highly effective antioxidant that mainly contributes to cellular defense against oxidative stress. Its significant properties include a very high catalytic rate of reaction and high stability under physico-chemical stress [40]. SOD is classified into four different types based on its metal centers, including Cu, Zn-, Fe-, Mn-, and Ni [41]. Cu, Zn-SOD (SOD1) is primarily found in the cytosolic and lysosomal fractions, but it is also present in the mitochondrial intermembrane space. Mn-SOD (SOD2) is in the mitochondrial matrix. Both Cu, Zn-SOD and Mn-SOD are abundant in neural tissue [42].

### 2.5. Catalase (CAT)

Catalase plays an essential role in a versatile response to H_2_O_2_ and is strategically positioned as an auxiliary to GPx. It serves as a second-tier defense against ROS. Naturally, peroxisomes contain crystalline inclusions of catalases and exhibit prominent antioxidant functions [43]. Catalases are classified as monofunctional, bifunctional, and pseudo-catalase. Monofunctional catalases are mainly reported with similar molecular features across animals, plants, fungi, and bacteria. They exhibit minor peroxidase activity, targeting small organic substrates. Catalase’s function across a broad range of pH levels (5–10), are resistant to organic solvents since they are made up of glycoproteins, and their activity can be inhibited by 3-amino-1,2,4-triazole [44].

### 2.6. Neuroinflammation and Its Role in Neuronal Damage

**Figure 3:**
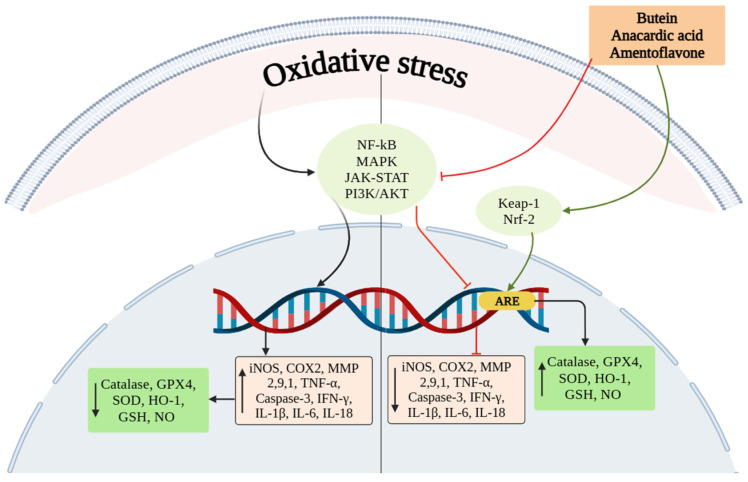
Oxidative stress activates signaling molecules such as NF-ĸB, MAPK, JAK/STAT, and PI3K/AKT that ultimately upregulates the expression of inflammatory mediators and downregulates enzymatic antioxidants. Meanwhile, treatment with SAE and its phytocomponents induce the expression of Nrf2 and inhibits NF-ĸB expression. Nrf2 expression upregulates the level of enzymatic antioxidants and reduces inflammatory mediators.

Oxidative stress and inflammation are cognitive players of programmed cell death [45,46]. As shown in Figure 3, oxidative stress reduces the levels of enzymatic antioxidants and increases ROS, which ultimately triggers the expression of inflammatory genes, pro-inflammatory mediators and pro-apoptotic genes.

**Figure 4:**
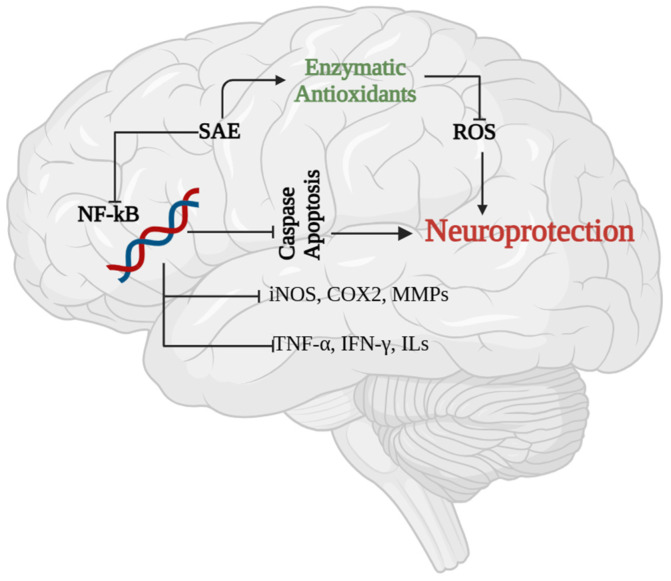
Neuroprotective effect of SAE. The expression of NF-ĸB is higher in inflamed neuron cells, which regulates apoptosis through activation of caspases. Treatment with SAE and its phytocomponents were shown to inhibit NF-ĸB, caspase expression and pro-inflammatory mediators. Meanwhile SAE treatment was shown to enhance enzymatic antioxidants in neuron cells that reduces the level of ROS and protects neuron cells from apoptosis.

Pathological states such as glutamate excitotoxicity, bacterial or viral infections, ischemic or haemorrhagic stroke, or oxidative stress to the CNS or brain cause initial injury and lead to cytokine-mediated inflammation and activation of inflammatory genes. These stimuli activate either canonical or non-canonical NF-κB pathways, which have been implicated in neuroinflammation-related pathogenesis [47]. Inducing the expression of these genes results in the activation of apoptotic proteins and ultimately causes cell death (Figure 4).

### 2.7. NF-κB and Neuronal Inflammation

NF-κB expression plays a crucial role in inflammation, immune responses [48], cell cycle, and cell survival [49]. NF-κB is a ‘master immune regulator’ [48] and a member of the Rel family of transcription factors. In mammals, p65 (RelA), RelB, c-Rel, p50/p105 (NF-κB1), and p52/p100 (NF-κB2) are five distinct NF-κB family members that share similar amino acid sequences [50]. The term NF-κB refers to dimers composed of any combination of two transcription factors from this family. NF-κB is mostly present in the cytoplasm until it is activated by pro-inflammatory stimuli [48]. IκB degradation induces the release of NF-κB, which is ubiquitously expressed in neurons, glial cells, and cerebral blood vessels, and regulates the transcription of chemokines, cytokines, adhesion molecules, proinflammatory enzymes, and transcription factors within the neuronal environment [47].

## 3. Effect of *S. anacardium* L. Crude Extract on Neuronal Oxidative Stress and Damage

### 3.1. Role of SAE on Antioxidant Genes

The flavonoids purified from *S. anacardium* L. were shown to increase the expression of genes p38 and Nrf2, as well as enzymes like catalase and SOD, to counteract oxidative stress [51]. *SAE*-mediated inhibition of NF-ĸB and AP-1 suppressed the LPS-induced expression of pro-inflammatory cytokines (IL-1β, IL-12p40, and NO) [52]. LPS is a classical TLR4 ligand [53] that stimulates MAPK family members of the monocyte lineage, induces the expression of NF-ĸB, AP-1, Sp1, C/EBPβ, Spi-1, and interferon regulatory factors [52], and provokes the induction of inflammatory genes including MMPs [54], iNOS [55], and COX-2 [56].

LPS induced cognitive impairment and neuroinflammation in C57BL/6J mice, confirmed through immunofluorescence, ELISA, and Western blot. LPS increased the expressions of COX-2 and iNOS in brain homogenates [57]. Biflavonoids from *S. anacardium* L. have been reported to inhibit COX-1 and COX-2 in a dose-dependent manner in vitro [58]. SAE activates the Nrf2/Keap1 signaling axis, leading to transcriptional upregulation of antioxidant enzymes and restoration of cellular redox balance. Oxidative stress indicators, including LDH leakage, increased caspase-3 levels, mitochondrial dysfunction, and DNA damage, were reversed by SAE. The potential of SAE to increase GSH levels by affecting the redox state [59] was demonstrated in vivo using models of mammary carcinoma [27] and DL [60].

### 3.2. Recovery of Neuronal Function by SAE

SAE reversed ultrastructural changes in the hippocampal neuron cell bodies of rats subjected to long-term immobilization stress, affecting both pyramidal and granule cells. Treatment decreased the number of degenerating cell bodies; this process is linked to increased corticosteroid levels and free radical production. Hippocampal tissues are a target for glucocorticoids, which may lead to neurodegeneration. SAE significantly reduced glucocorticoid levels and was followed by stress treatment [61]. A similar effect was observed in Wistar albino rats with AlCl_3_-induced dementia [62]. β-amyloid plaques disrupt cell homeostasis and increase calcium ion levels in mitochondria and cytosol, which together lead to excess ROS levels [63]. SAE enhanced learning and reduced toxicity-related memory problems. Additionally, cholinesterase activity and amyloid plaque build-up in the brain decreased after treatment with SAE [62].

SAE has been reported to improve spatial memory by inhibiting acetylcholinesterase, reducing oxidative stress, and preventing glutamate-induced calcium influx [64]. The neuroprotective properties of SAE were demonstrated in NH_4_Cl-induced hyperammonemic rats [65] and in L-monosodium glutamate-treated in vitro and in vivo models. Glutamate is essential for rapid responses to stimuli and neurological functions such as cognition, memory, movement, and sensation [66]; however, excessive levels of glutamate can cause brain damage by hyperactivating ionotropic glutamate receptors through the excitotoxicity pathway [67].

Glutamate-induced oxidative stress occurs through various mechanisms, leading to glutathione depletion, increased Ca^2+^ levels, excessive ROS production, and inhibition of cystine uptake [68]. SAE has been shown to protect hippocampal neurons in albino rats [61] and male Wistar rats treated with high doses of L-monosodium glutamate (4 g/kg) [69]. The strong interactions and conformational stability of *S. anacardium* L. derivatives with AD targets such as AChE [70,71], NMDA, TTBK1, and BACE-1 have been reported in silico.

Experimental evidence strongly supports the role of SAE on activating antioxidant genes and recovering neural function. The various methods of extraction, key phytochemicals, and molecular targets are represented in Table 1.

Collectively, these findings indicate that SAE exerts neuroprotective effects through integrated modulation of oxidative stress, inflammatory signalling, and apoptosis.

## 4. The Role of Butein in Neuronal Damage

Butein is a naturally occurring plant-derived metabolite, first extracted from *Toxicodendron vernicifluum*, formerly known as Rhus verniciflua [72], and its presence has also been reported in *Semecarpus anacardium*, *Dalbergia odorifera*, and the flowers of *Butea monosperma* [73]. The molecular weight, toxicological and pharmacological properties of butein are illustrated in Table 2.

### 4.1. Butein Activates Nrf2/ARE Pathway

Nrf2 expression is linked to neurological disorders because its deficiency causes mitochondrial failure, oxidative stress, and neuroinflammation [84]. Butein promotes the upregulation of the Nrf2/ARE signaling pathway, which depends on PI3K/AKT activation and increases HO-1 levels to exert anti-neuroinflammatory effects in HT22 and BV2 microglia cells [85]. Butein significantly reduces glutamate-induced cell death and ROS production in HT22 cells by increasing HO-1 levels, a key component of the antioxidant system. The translocation of Nrf2 regulates various antioxidant genes, leading to ARE-mediated induction of phase-II detoxifying enzymes, including HO-1, catalase, glutathione, glutathione-S transferase, glutathione reductase, and glutathione peroxidase [86]. The transcription factor Nrf2 activation counters oxidative stress through its endogenous inhibitor Keap1 and reduces cellular damage [87]. Additionally, inhibition of Nrf2 by siRNA or trigonelline results in increased neuroinflammation in BV2 microglia cells. Butein-induced expression of HO-1, which reduces oxidative stress via Nrf2, was shown in vitro [88,89] and in vivo [90]. Nrf2 binding to ARE can activate downstream antioxidant enzymes, including HO-1, GSH, NQO-1, and GST. These enzymes help lower ROS levels and protect cells and tissues.

Corticosterone is a glucocorticoid stress hormone linked to several neuronal disorders. Butein countered corticosterone-induced oxidative stress-related toxicity in mouse neuroblastoma Neuro2A (N2A) cells. Corticosterone caused apoptosis through mitochondrial dysfunction, caspase-3 activation, and ROS production. Butein significantly lowered ROS levels, LDH leakage, caspase-3 activity, mitochondrial potential loss, and DNA damage [91].

### 4.2. Butein Suppressess Transcription Factor NF-ĸB

NF-ĸB and the MAPK family (JNK, p38, and ERK) are widely distributed in cells and tissues, playing a crucial role in neuroinflammation, and their signaling is upregulated in SH-SY5Y cells. Phosphorylation of the tau protein promotes neuroinflammation through the ERK pathway, which leads to the activation and nuclear translocation of NF-ĸB [92].

The activity of NF-ĸB was inhibited by pretreatment with butein, which increased the viability of SH-SY5Y cells grown in conditioned medium from BsV2 microglial cells [93]. Activated microglia contain high levels of intracellular ROS that cause oxidative damage and inflammatory conditions [94], where NF-ĸB mediates the secretion of pro-inflammatory cytokines [94], leading to chronic inflammatory reactions [95] and resulting in neuronal damage or death [96].

Butein demonstrated its neuroprotective effects by inhibiting the NF-ĸB signaling pathway in microglial cells [85,93] and in a traumatic spinal cord injury model [18]. Cytotoxic factors released from BV2 microglial cells reduced SH-SY5Y neuronal viability before exposure to Butein. TLR4 mediated BV2 microglial activation and induced secretion of NO, PGE2, iNOS, COX-2, and pro-inflammatory cytokines [97]. This activation leads to neuronal synaptic dysfunction and neuronal death [98]. Studies on HT22 cells showed that butein reduces neuroinflammation by decreasing NO and PGE2 secretion and lowering inducible NOS and COX-2 expression via the NF-ĸB signaling pathway [85].

### 4.3. Effect of Butein on SCI

SCI is a serious spinal cord complication that causes significant dysfunction in the affected area. The inflammatory response is crucial in SCI development, involving key cell types such as macrophages, endothelial cells, microglia, and astrocytes [99]. The IKK/NF-κB signaling pathway plays a critical role in controlling inflammation and cell death, impacting SCI pathology. Targeting this pathway is a promising approach to improve locomotor recovery, decrease immune cell infiltration, and minimize apoptosis in rats following SCI. Butein reduces the IKK/NF-ĸB pathway, as shown in vivo. Traumatic SCI damages neural structures and leads to neurological deficits, with NF-ĸB signaling involved in secondary SCI damage [18]. Butein inhibited the IKK/NF-ĸB pathway and decreased apoptotic protein expression in spinal cord tissue. Apoptosis is a key factor in secondary SCI damage [100]. Neuronal and oligodendrocyte apoptosis triggers caspase-3 expression, causing axonal degeneration and loss of neural function [101,102]. Butein mitigated the activation of the IKK/NF-ĸB pathway and reduced inflammatory cell infiltration and caspase-3 activation in the spinal cords of Sprague-Dawley rats with SCI within 24 h [18]. Inflammation significantly contributes to SCI, with NF-ĸB being the main transcriptional regulator of inflammatory genes [103]. The IKK/NF-ĸB cascade plays a central role in regulating inflammation and apoptosis [18].

### 4.4. Role of Butein on MMP Expression

Butein reduced expression of genes including Bcl-2 [104], c-Myc [105], and COX-2 [106]. Bcl-2 enhances the production of inflammatory mediators, making control of Bcl-2 expression a key strategy for managing inflammatory and allergic reactions. Bcl-2 inhibitors have been reported to prevent airway inflammation [107], human tubulointerstitial inflammation [108], and experimental allergic rhinitis [109].

Butein significantly reduced IL-1β-induced inflammatory reactions in human and mouse osteoarthritis models. It suppressed the expression of COX-2, iNOS, TNF-α, IL-6, MMP-1, MMP-3, and MMP-13 [110]. In the CNS, MMPs are shown to degrade basal laminae components, leading to BBB disruption and contributing to neuroinflammation. In response to cellular stress, brain cells produce both constitutive and inducible MMPs [111]. Transcriptome analyses [112] and patient data [113] demonstrate the link between MMP-9 expression and inflammatory diseases. Therefore, natural and synthetic MMP inhibitors are emerging as treatments for inflammatory conditions [114]. NF-ĸB-mediated suppression of MMP-9 was shown in vitro with Butein [105]. SAE exemplified the normalization of MMP-1, MMP-2, MMP-3, TIMP-1, and TIMP-2 levels in a mammary carcinoma model [115]. The presence of flavonoids in the extract may enhance membrane stability and prevent lysosomal hydrolase secretion [116,117]. Lysosomal activity plays a critical role in MMP-9 secretion and the inflammatory response [118].

**Figure 5:**
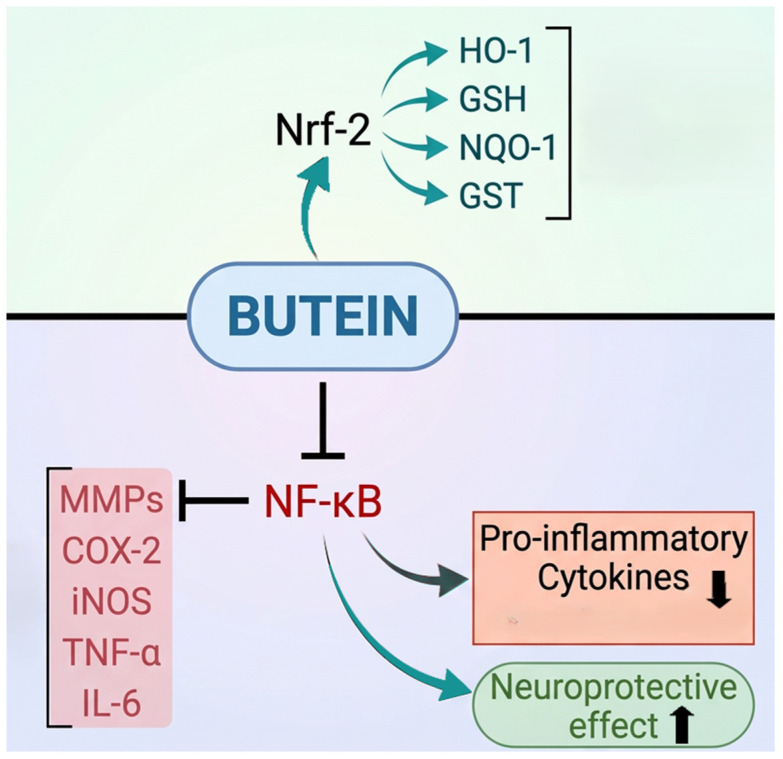
Butein increases Nrf2 expression, which boosts antioxidant enzyme levels that counteract oxidative stress. At the same time, butein decreases NF-κB expression, leading to lower levels of pro-inflammatory cytokines and related factors.

The dual role of butein in activating antioxidative enzymes and reducing inflammation is supported by various experimental studies. In summary, Figure 5 illustrates the butein mediated upregulation of Nrf2, which enhances the expression of antioxidative enzymes. Meanwhile, butein downregulates NF-ĸB expression, leading to a reduction in pro-inflammatory mediators. Thus, butein exerts dual regulatory control over oxidative stress and neuroinflammation through coordinated modulation of antioxidant–inflammatory signaling interplay. Table 3 summarizes the molecular targets and mechanism of action of butein.

## 5. Anacardic Acid and Neuroinflammation

The presence of phenolic, carboxylic, and a 15-carbon alkyl side chain functional group makes it desirable for biological applications. Anacardic acid serves as a potential starting material for synthesizing various biologically active compounds [119]. The toxicological and pharmacological properties of anacardic acid are illustrated in Table 4.

### 5.1. Role of Anacardic Acid on Enzymatic Antioxidants

Anacardic acid modulates redox homeostasis by enhancing antioxidant enzyme expression while concurrently suppressing oxidative enzyme activity [126]. Anacardic acid was also found to elevate GSH levels in the prefrontal cortex and hippocampus of mice following acute intraperitoneal doses of 25 and 50 mg/Kg. This indicates that the effect was consistent across different brain regions, doses, and methods of administration [127]. Anacardic acid increased overall SOD gene expression in both mitochondria and the cell cytoplasm. Treatment with anacardic acid increased the expression of GPx4 [128] that regulates lipid peroxidation and inflammatory cytokines. The GPx4^−/−AT^ mouse model demonstrated the protective role of GPx4 against systemic low-grade inflammation [129].

Moreover, GPx4 plays a crucial role in iron-dependent cell death [116,130]. TBI-mediated inhibition of GPx4 expression results in ROS accumulation, redox imbalance, and ferroptosis induction [131,132] TBI is a severe cause of neuronal disability that leads to iron-dependent lipid peroxidation and oxidative cell death [133]. It is characterized by the pathophysiological processes of edema, inflammation, ferroptosis, and programmed cell death. Anacardic acid mitigates ferroptosis by upregulating GPx4 expression, reducing lipid peroxidation, and limiting iron-dependent oxidative damage. It lessens ferroptosis severity by reducing inflammation and oxidative stress and by modifying the expression of key ferroptosis proteins. Furthermore, anacardic acid decreases iron deposition in tissues as part of its anti-ferroptosis effects and enhances BBB permeability [128].

### 5.2. Protective Effect of Anacardic Acid on PD

Anacardic acid exerts a protective effect against PD [127,134]. Oral administration of anacardic acid demonstrates preventive antioxidant activity in the rat nigrostriatal system and cerebral cortex in a pesticide rotenone-induced experimental model of PD. Anacardic acid completely blunted rotenone-provoked lipoperoxidation [127,134] by stimulating t-SOD [134]. Additionally, it increased NO levels and reduced the redox balance of GSH/GSSG in the substantia nigra and striatum [127]. There was a significant increase in the gene expression of both SOD-1 and SOD-2 in the striatum, with 2490- and 190-fold increases, respectively [134]. SOD is predominantly expressed in glial cells and throughout the CNS. Clinical and genetic evidence shows a direct correlation between SOD gene mutations and neurodegenerative diseases [135].

Furthermore, the protective effect of anacardic acid was demonstrated in a pesticide rotenone-induced experimental model of PD in Wistar rats. Sufficient amounts of anacardic acid crossed the BBB, reached the CNS, and exerted beneficial effects. An increase in SOD gene expression and t-SOD activity confirmed the neuroprotective effect of anacardic acid [134]. The results support the findings of Augusto et al. on PD induced in Swiss mice [127].

Neuro histological and neuroimaging studies have shown ongoing and end-stage neuroinflammatory processes in PD. Samples of peripheral blood and cerebrospinal fluid from PD patients reveal variations in inflammation markers and immune cell populations that can worsen neuroinflammation and contribute to neurodegeneration [136]. Oxidative stress causes cellular damage, triggers NF-ĸB expression, and activates inflammatory processes that lead to cytokine secretion in neurodegenerative diseases such as PD [137,138].

### 5.3. Regulation of Anacardic Acid on Inflammatory Mediators

Anacardic acid demonstrates anti-inflammatory effects by inhibiting anti-oxidative enzymes, pro-inflammatory mediators, and NF-ĸB. It notably lowers TNF-α- induced mRNA levels of NF-κB, a nuclear transcription factor associated with inflammation. Suppressing NF-κB diminishes free oxygen radicals and reduces oxidative stress [139]. In a carrageenan-induced peritonitis model, anacardic acid lowered total leukocyte and neutrophil migration and reduced MPO activity. In neutrophils, MPO is crucial for activating p38 MAPK and NF-κB pathways. MPO also stimulates the production of cytokines such as IL-6 and IL-8, as well as ROS [140]. Anacardic acid has been shown to reduce pro-IL-1β levels in the striatum [127] and inhibit the gene expression and activity of MMP-2 and MMP-9 [141]. MMP-9 supports glial activation and neurodegeneration in both monkey and mouse models of PD induced by MPTP or rotenone [142,143]. Anacardic acids were also shown to decrease MMP-9 and increase TIMP-1 protein levels in vivo [127] Phenolic compounds were shown to downregulate inflammatory markers in various brain regions [141].

### 5.4. Anacardic Acid vs. Apoptotic Molecules

The role of apoptotic molecules in inflammation depends on their substrates. proIL-1β and proIL-18 are key in inflammation and serve as substrates for caspase-1 [144]. The participation of caspases in apoptosis, pyroptosis, and necroptosis is a shared characteristic. Caspase-driven proteolysis causes either gain-of-function or loss-of-function effects on their substrates, leading to inflammation [145]. Anacardic acid was shown to reduce caspase-3 levels while increasing the expression of Bcl-2 [146]. Caspases, activated by TNF-α, initiated programmed cell death by destroying critical components of the cellular infrastructure that mediate cell damage [147]. Bcl-2 family members can be either pro-apoptotic or anti-apoptotic, and their balance depends on the release of cytochrome C [148]. However, caspases can also influence the homeostasis of pro-apoptotic or anti-apoptotic signals from the Bcl-2 family [149].

### 5.5. Anacardic Acid vs. Voltage-Gated Ion Channels

Intracellular Ca^2+^ homeostasis regulates neurodegenerative processes [150], where SAE treatment normalized intracellular Ca^2+^ levels in PC-12 cells [69]. Reduced levels of ROS, calcium influx, toxicity, and increased cell viability were observed in PC-12 cells treated with bhilawanol and anacardic acid. Calcium entry across the cell membrane is ROS-dependent [151], and higher calcium entry was significantly decreased by the treatment. Bhilawanol and anacardic acid are lipid-soluble compounds found in *S. anacardium* L. which potentially inhibit AChE in a dose- and time-dependent manner. In addition, a glutamate receptor antagonist and AChE inhibitors are FDA-approved drugs for AD [146].

Furthermore, the neuroprotective activity of anacardic acid has been demonstrated in an experimental epilepsy model. An imbalance in the function of oxygen- or nitrogen-derived reactive species affects brain activity, leading to mitochondrial dysfunction, DNA damage, changes in neural signalling, and hindrance of neurogenesis [152] Oxidative stress-mediated disruption of calcium signalling, mitochondrial impairment, and cellular damage result in neuronal cell death, seizures, and systemic toxicity [153]. Anacardic acid reduced epileptic seizures in a dose-dependent manner, potentially through inhibition of Na+ voltage-dependent channels [125] The mechanism of action of anacardic acid and its molecular targets are listed in Table 5.

This coordinated regulation of ferroptosis, oxidative stress, and inflammation highlights anacardic acid as a multifunctional neuroprotective agent.

## 6. Amentoflavone

Amentoflavone belongs to the class of biflavonoids and polyflavonoids known as 3′, 8″-biapigenin [154]. The toxicological and pharmacological properties of amentoflavone illustrated in Table 6.

### 6.1. Amentoflavone vs. Antioxidants

Amentoflavone reduces oxidative stress by enhancing Nrf2-mediated antioxidant responses while simultaneously suppressing TLR4/NF-κB-driven inflammation. It alleviates oxidative stress and neuroinflammation-induced cerebral ischemia/reperfusion in rats via the TLR4/NF-κB signaling pathway. Pro-inflammatory cytokines are involved in the body’s inflammatory response to traumatic brain injury. Treatment with amentoflavone significantly decreases serum levels of TNF-α, IL-1β, and IL-6 compared to the control group [157]. Low levels of catalase, SOD, and GPx limit the brain’s ability to eliminate superoxide anion and hydrogen peroxide, making brain tissue more vulnerable to ROS and oxidative stress [167]. Amentoflavone significantly raised brain antioxidant markers GSH and CAT and lowered MDA levels [157].

Amentoflavone activates AMPK-dependent Nrf2 signaling, promoting transcription of antioxidant enzymes and protecting against Aβ-induced neurotoxicity. In transgenic AD mice, the Nrf2 pathway was compromised, alongside increased brain Aβ levels. Treatment with amentoflavone significantly boosted Nrf2 expression and its translocation, which in turn increased HO-1 and NQO-1 levels, thereby reducing oxidative stress in the brain. Suppressing Nrf2 expression in neuronal cells weakened amentoflavone’s protective effects, indicating that Nrf2 plays a critical role in its neuroprotective activity [168].

Its neuroprotective role was demonstrated in transgenic ADAT/Nrf2-KO mice, where accumulation of Aβ and p-tauS404 was observed in its absence [169]. Additionally, amentoflavone inhibited ferroptosis-mediated inflammation via the SLC7A11/GPx4 axis in homocysteine-induced neuronal dysfunction. Homocysteine-exposed HT22 cells showed increased mRNA levels of ferroptosis-related genes and iron accumulation [170].

### 6.2. Amentoflavone Influences the Expression of NF-ĸB

The role of amentoflavone in fighting stroke was shown with cerebral ischemia/reperfusion (IR) injury in unilateral common carotid artery occlusion (CCAO). Amentoflavone increased GSH and CAT activities and reduced neuroinflammation by negatively regulating the TLR4/NF-ĸB signaling pathway, providing neuroprotective benefits [157]. Proinflammatory cytokines are known mediators of brain damage after HI injury. Yang et al., noted that elevated systemic and cerebral IL-6 levels are part of traumatic brain injury and inflammation [171].

In addition to inhibiting the TLR4/MyD88/NF-ĸB cascade, amentoflavone activated the Nrf2/HO-1 pathway in LPS-induced BV2 microglia cells and reversed neuroinflammation [172]. TLR4-mediated inflammation is a key factor that contributes to neurodegeneration in PD [173]. Prior research supports the role of amentoflavone in blocking the TLR4/MyD88/NF-κB signaling pathway [174].

Administering amentoflavone to rats with ischemic injury reduced serum cytokines (TNF-α, IL-1β, and IL-6) compared to the control. A lower level of the TBK1 pathway is linked to neuroinflammation, which plays a key role in selective autophagy and inflammatory IFN signalling. Similarly, NF-ĸB is important in the secretion of iNOS synthetase and inflammation—brain cells such as astrocytes, microglia, and oligodendrocytes exhibit activated NF-ĸB complexes. Amentoflavone treatment significantly decreased NF-ĸB expression and improved TBK1 and IFN-β function [157].

The strong neuroprotective effect of amentoflavone is shown by improving neurological function, increasing motor coordination, and boosting locomotor activity. Amentoflavone reduced ischemia/reperfusion-induced brain injury through the HMGB1-mediated TLR4/NF-ĸB signalling pathway in a unilateral common carotid artery occlusion (CCAO) rat model. IR control rats showed hemorrhage with pyknotic nuclei in neurons of the cerebrum. In contrast, brain sections from treated animals displayed mild congestion of cerebral blood vessels, along with some vacuolar degeneration in neurons and perivascular edema [157].

Amentoflavone inhibited the activation and nuclear translocation of the NF-ĸB subunit p65. As a result, amentoflavone suppressed inflammation, apoptosis, and prevented the excessive discharge of hippocampal neurons, thereby avoiding pilocarpine-induced epilepsy [175]. Flavonoids have been reported to interact with MAPK signalling pathways that regulate various cellular functions [176]. Amentoflavone increased the expression of PI3K and Akt, as well as the Bcl-2/Bax ratio, and protected against the progressive degeneration of dopaminergic neurons [177] in vitro, amentoflavone treatment reduced nuclear condensation and cell viability loss induced by 1-methyl-4-phenylpyridinium.

### 6.3. Amentoflavone Controls HI Injury

Similar research supports the role of amentoflavone in protecting against hypoxic-ischemic damage in rat brains. Administering amentoflavone systemically 3.5 h after HI injury offers strong neuroprotection in neonatal cases. Its dual function of reducing cytotoxic damage and providing anti-inflammatory effects enhances outcomes in HI injury [166]. Amentoflavone reduces microglial inflammation after HI injury. It blocks the upstream cell death cascades that cause both necrotic and apoptotic neuronal death following HI injury. The presence of the active form (p18) of caspase 3 indicates that amentoflavone inhibits caspase 3 activation [166]. Brain proinflammatory cytokines can mediate brain damage after HI injury. For example, microglia, astrocytes, and neurons secrete early response cytokines such as IL-1β and TNF-α [178].

Amentoflavone inhibits the induction of proinflammatory mediators, including iNOS, COX-2, IL-1β, and TNF-α, in microglial BV-2 cells. Amentoflavone protected hippocampal neurons in epilepsy mice by reducing inflammation and preventing cell death. It also decreased the gene expression of inflammatory mediators like IL-1β and iNOS in the substantia nigra pars compacta of MPTP-induced mice. Additionally, amentoflavone significantly reduced OX-42 immunoreactivity, a biomarker for activated microglia and LPS-induced worsening of HI brain damage, supporting the neuroprotective effects of amentoflavone in neonatal HI injury [166]. Hampered caspase-3 and p21 function and increased Bcl-2/Bax expression were observed with amentoflavone treatment in SY5Y cells [177]. The role of amentoflavone on various neurological conditions, its molecular target, and reported concentrations were listed in Table 7.

Overall, amentoflavone exerts neuroprotective effects through coordinated modulation of oxidative stress, inflammatory signaling, and ferroptosis pathways.

## 7. Mechanism of Action of *S. anacardium* L. and Its Phytocompounds

**Figure 6:**
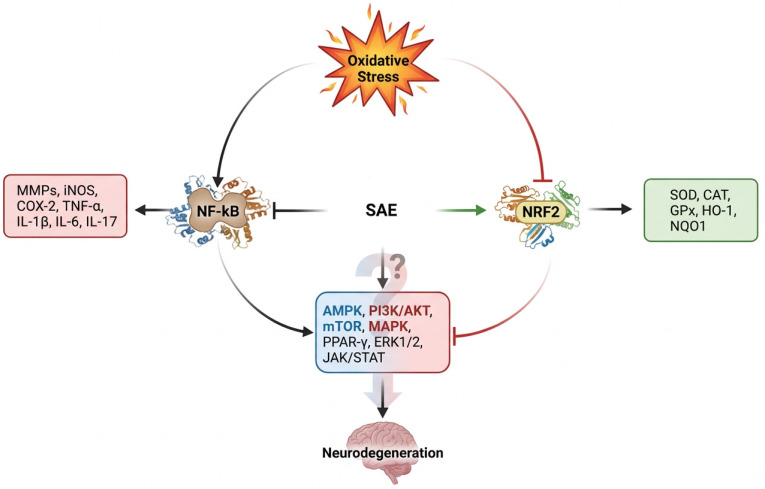
Mechanistic overview of SAE-mediated modulation of oxidative stress and inflammatory pathways in neurons. Extensive evidence supports the oxidative stress mediated upregulation of NF-ĸB and inflammation where the levels of Nrf2 reduced with oxidative stress. SAE and its phytocomponents were shown to inhibit NF-ĸB expression and activation of Nrf2. Higher expression of Nrf2 elevates the level of enzymatic antioxidants which protect the cells from neurodegeneration. Consequently, reduced level of pro-inflammatory mediators was observed with NF-ĸB inhibition. However, the role of SAE and its phytocomponents on the regulation of transcription factors, such as AMPK, PI3K/AKT, mTOR, MAPK, PPAR-γ, ERK1/2, and JAK/STAT needs to be explored.

Studies have demonstrated that SAE activates the Nrf2/Keap-1 signaling pathway, thereby enhancing the expression of endogenous antioxidant enzymes under conditions of oxidative stress and inflammation (Figure 6). In addition, *S. anacardium* extract (SAE) and its bioactive compounds have been reported to suppress NF-κB signaling, resulting in the downregulation of key inflammatory mediators, including MMPs, iNOS, and COX-2. Furthermore, several intracellular signaling cascades such as AMPK, PI3K, AKT, mTOR, MAPK, PPAR-γ, ERK1/2, and JAK/STAT are modulated in response to NF-κB activation status and play critical roles in the progression of neuroinflammation. However, direct evidence elucidating the interaction between *S. anacardium* extracts or their phytoconstituents and these molecular pathways remains scarce and warrants further investigation. Phytochemical classes including alkaloids, terpenoids, flavonoids, saponins, glycosides, and steroids substantially contribute to the therapeutic potential of medicinal plants. Notably, the seeds of *S. anacardium* extracts are rich in phenolic compounds and flavonoids, which are primarily responsible for their strong antioxidant capacity [16]. Polyphenols have been widely reported to attenuate oxidative stress through activation of Nrf2 signaling [179,180], enhancement of antioxidant defense systems [181], and inhibition of NF-κB–mediated inflammatory pathways [182]; Comparable mechanistic effects have also been observed for specific compounds such as butein, anacardic acid, and amentoflavone. As a crude extract, SAE is likely to exert pleiotropic effects by targeting multiple signaling pathways simultaneously [183]; however, such multi-target actions have not yet been conclusively validated through experimental studies. In addition, growing evidence suggests that synergistic interactions among dietary phytochemicals may further enhance their efficacy in mitigating oxidative stress and promoting neuroprotection [184]. Variability in extraction methods and solvent systems significantly influences the phytochemical composition of SAE, thereby altering biological activity. Standardization of key bioactive compounds such as butein, anacardic acid, and amentoflavone remains essential for reproducibility and clinical translation.

## 8. Future Perspective

This review highlights the neuroprotective potential of *SAE* and its key phytocomponents-butein, anacardic acid, and amentoflavone-in modulating oxidative stress and neuroinflammation, which are central drivers of neurodegenerative diseases. Collectively, these compounds exert dual regulatory effects by activating Nrf2-mediated antioxidant pathways while concurrently suppressing NF-κB-driven inflammatory signaling. Despite robust preclinical evidence, several limitations hinder translational advancement. Variability in extract composition, lack of standardized formulations, insufficient pharmacokinetic and ADMET profiling, and limited in vivo studies remain critical challenges. Furthermore, most studies rely on acute or simplified disease models that do not fully capture the complexity of human neurodegenerative disorders. Future research should focus on standardized extract characterization, mechanistic validation using multi-omics approaches, and evaluation in clinically relevant models. Integration of advanced delivery systems to improve bioavailability, along with well-designed clinical studies, will be essential to translate these promising phytocompounds into therapeutic interventions. Collectively, these findings position SAE and its phytocompounds as a promising multi-target therapeutic candidate, bridging traditional medicine and modern neuropharmacology.

## Figures and Tables

**Table 1:** Overview of *Semecarpus anacardium* L. extracts/compounds detailing origin, method of extraction, key phytochemicals, experimental concentrations and observed cellular and systemic effects.

Source	Solvent and Method of Extraction	Phyto Chemicals	Concen Tration	Mechanism of Action	Reference
Stem bark	Methanolic extract applied to silica gel column	3-O-methyl quercetin, Kaempferol	100 μg/mL	Exhibited cytoprotective and antioxidant activity by reducing ROS, preventing cellular and DNA damage, and upregulating Nrf2 mediated antioxidant defense	[51]
Nut	Ethanolic extract-Maceration method	crude	1mg/mL	Exhibited anti-inflammatory activity by inhibiting IL-1β & IL-12p40, suppressing NF-κB/AP-1 signalling and NO production without affecting TNF-α and IL-6	[52]
Seeds	Hexane, chloroform, ethyl acetate and methanol; Silica gel chromatography	Tetrahydro amentoflavone	100 μg/mL	Exhibited potent anti-inflammatory activity via COX inhibition and significant reduction of edema comparable to ibuprofen	[58]
Nut	Aqueous extract-decoction method	crude	6 mg/mL per mouse	Exhibited antioxidant and anticarcinogenic activity by enhancing antioxidant enzymes and reducing LDH levels, indicating suppression of tumor progression	[60]
Nut	Milk extract-decoction method	crude	200 mg/kg/body weight	Exhibited antioxidant and chemoprotective activity by restoring GSH redox balance and normalizing glutathione-dependent enzymes under carcinogenic condition	[27]
Nut	Chloroform, Chloroform methanol and ethyl alcohol extract-Soxhlet extraction method	crude	40 mg/kg/body weight	Conferred marked neuroprotection by attenuating stress-induced neuronal degeneration and preserving hippocampal cellular integrity	[61]
Nut	Milk extract-decoction method	crude	150 mg/kg/body weight	Ameliorated hyperammonemia-driven neurotoxicity by rebalancing oxidant-antioxidant status, normalizing metabolic and hepatic markers, and preserving neuronal integrity	[65]

**Table 2:** Toxicological and pharmacokinetic profile of butein.

Phyto Compound	Lethal Dose	Toxicological and Pharmacokinetic Observations	Reference
Butein	10–100 mg/kg/day (i.p. or oral), 21 days	Well-tolerated in animal models; no mortality at therapeutic doses. No defined LD_50_ or sub-chronic data	[74]
	50 mg/kg/day (oral), 14 days	No cardiac or systemic toxicity observed at these concentrations	[75]
	75 mg/kg/day (oral), 14 days	No hepatotoxicity or nephrotoxicity notes	[76]
	25, 50, 100 mg/kg/day (oral), 14 days	No behavioral toxicity; preserved neuronal morphology	[77]
	50, 100 mg/kg/day (oral), 14 days	Histopathology confirms preserved hepatic and renal architecture	[78]
	10, 20, 40 mg/kg/day	No visible toxic signs or mortality	[79]
	10, 15, 20 mg/kg/day	No systemic or behavioural toxicity observed	[80]
	-	Requires standardization of extract, GLP-compliant chronic toxicity, and PK/ADME studies for clinical validation	[81,82,83]

**Table 3:** Molecular targets and mechanisms of butein in neuronal inflammation.

Phyto Compound	Disease or Condition	Target	Concen Tration	Mechanism of Action	Reference
Butein	Glutamate-induced neurotoxicity & LPS-induced neuroinflammation	NF-κB, Nrf2/HO-1; PI3K/Akt; IL-6/IL-1β/TNF-α; iNOS/COX-2	10 μM	Attenuates oxidative neuronal injury and inflammatory activation by enhancing Nrf2/HO-1 mediated cytoprotection and suppressing NF-κB driven pro-inflammatory signalling	[85]
	CORT induced neurotoxicity (Neuro2A cells; depression model)	Mitochondrial dysfunction; caspase-3 activation	0.5 μM	Butein shields Neuro2A cells from CORT-induced apoptosis by preventing mitochondrial dysfunction, caspase-3 activation, and DNA damage	[91]
	Neuroinflammation (LPS activated microglia- conditioned SH-SY5Y cells)	NF-κB p65; ERK signalling; apoptosis	30 μg/mL	Shields neurons from microglia driven inflammatory injury by suppressing NF-κB activation and ERK signalling, thereby reducing apoptosis and improving cell survival	[93]
	Spinal cord injury (SCI)	IKK/NF-κB; IκB; caspase-3	0.5 mg/mL	Limits post-injury inflammatory and apoptotic cascades by inhibiting IKK/NF-κB signalling, reducing neutrophil infiltration and caspase-3 activation, thereby supporting functional recovery	[18]

**Table 4:** Illustrates the therapeutic outcomes, toxicological, and pharmacokinetic profile of phytocompound anacardic acid.

Phyto Compounds	Lethal Dose	Toxicological and Pharmacokinetic Observations	Reference
Anacardic acid	LD_50_ > 2000 mg/kg mice	No significant organ level, hematological, or biochemical changes observed at sub-acute doses ≤ 300 mg/kg. Low acute toxicity confirmed	[120]
	25, 50 & 100 mg/kg (oral, daily for 90 days)	No mortality, no dose–response toxicity in hematology, liver/metabolic profiles; minimal organ weight changes	[121]
	500 µg/kg, oral for 12 days	Anacardic acid reduces lipid levels	[122]
	-	Tumor volume/size reduced, lung metastasis decreased, immune cell infiltration improved, and side effects of Taxol reduced	[123]
	10, 25, 50 mg/kg, oral	Reduced immobilization time, comparable to imparine-treated controls, activity mediated partly through serotonergic and nitric oxide signalling pathways. No locomotor activity	[124]
	25, 50, 100 mg/kg, oral	Delayed seizure onset and reduced duration in PTZ and kainate models; partial protection in MES. Likely via GABAergic modulation and antioxidant effects	[125]

**Table 5:** Represents the therapeutic targets, molecular mechanism, and the concentrations reported for anacardic acid (↓ decrease; ↑ increase).

Phyto Chemical	Disease or Condition	Target	Concentration	Mechanism of Action	Reference
Anacardic acid	Parkinson’s disease (rotenone induced model)	NF-κB, IL-1β, MMP-9/TIMP-1	50 mg/kg	Neuroprotection via reducing oxidative stress (↓ LPO, ↓ NO, ↑ GSH/GSSG), suppression of inflammation (↓ NF-κB, ↓ IL-1β), and restoration of dopaminergic function (↑ TH)	[127]
	Traumatic brain injury (TBI)	Ferroptosis pathway	100 mg/kg/body weight	Neuroprotection via inhibition of ferroptosis and inflammation with improved neurological and cognitive function	[128]
	Parkinson’s disease (rotenone induced model)	t-SOD (Cytoplasmic and mitochondrial)	1–100 mg/kg/body weight	Dose-dependent mitigation of oxidative damage, enhanced mitochondrial antioxidant defense, and preservation of motor and cognitive performance	[134]
	Acute inflammation and nociception (mouse models)	MPO, MDA, GSH, opioid receptor	25 mg/kg/body weight	Suppresses inflammatory edema and leukocyte infiltration, reduces oxidative stress (↓ MPO, ↓ MDA, ↑ GSH) and alleviates pain behaviors via potential opioid receptor modulation	[140]

**Table 6:** Illustrates the therapeutic outcomes, toxicological, and pharmacokinetic profile of phytocompound amentoflavone.

Phyto Compounds	Lethal Dose	Toxicological and Pharmacokinetic Observations	Reference
Amento flavone	30, 50 mg/kg/day, intragastrical administration	No behavioral or systemic toxicity observed	[155]
	20, 40 mg/kg/day, oral	Demonstrated strong hepatoprotection without signs of hepatic or systemic toxicity; biochemical, hematological, and histopathological parameters remained within normal limits	[156,157]
	20, 40 mg/kg/day for 8 weeks	No behavioral or hepatic toxicity observed; improved hepatic steatosis and preserved pancreatic histoarchitecture at therapeutic doses	[57,158]
	50, 100 mg/kg/day, acute, 7–14 days	No mortality or histopathological abnormalities; normal organ weights and blood profiles	[159,160]
	100 mg/kg/day for 14 days	-	[161]
	40–100 mg/kg/day for 21 days	No behavioral or systemic toxicity observed	[162,163]
	40–100 mg/kg/day (oral or i.p) for 7 to 21 days	No behavioral or systemic toxicity observed	[164,165,166]

**Table 7:** Represents the disease, molecular targets, and concentrations of amentoflavone (↑ increase; ↓ decrease).

Phyto Chemical	Disease or Condition	Target	Concentration	Mechanism of Action	Reference
Amentoflavone	Cerebral ischemia/reperfusion (I/R) injury (stroke model)	HMGB1-TLR4/NF-κB; Caspase-3	40 mg/kg/body weight	Neuroprotective effect via antioxidant enhancement (↑ GSH, ↑ CAT), anti-inflammatory action (↓ cytokines), inhibition of HMGB1/TLR4/NF-κB signalling, and suppression of apoptosis (↓ caspase-3)	[157]
	Alzheimer’s disease (Aβ-induced model)	AMPK/GSK-3β/Nrf2 pathway	80 mg/kg/body weight	Neuroprotection via antioxidant activation (↑ Nrf2), reduced neuronal apoptosis, and improved cognitive function	[168]
	Hyperhomo cysteinemia-induced neuronal injury	SLC7 A11/GPX4axis; Ferroptosis pathway	10 μM	Neuroprotection via inhibition of ferroptosis (↓ iron, ↓ ptgs2/TFR1/Fth1), reduced oxidative stress (↓ ROS, ↓ MDA, ↑ GSH), and suppression of inflammation	[170]
	Neuroinflammation (LPS-induced microglial model)	TLR4/MyD88/NF-κB; Nrf2/HO-1	10 μM	Anti-inflammatory and antioxidant effect via inhibition of TLR4/NF-κB signalling and activation of Nrf2/HO-1 pathway	[172]
	Epilepsy (pilocarpine-induced model)	NF-κB	25 mg/kg/body weight	Neuroprotection via anti-inflammatory, antioxidant and anti-apoptotic effects with reduced seizures and neuronal loss	[175]
	Parkinson’s disease (MPTP/MPP^+^ model)	PI3k/Akt; ERK; Bcl-2/Bax; Caspase-3	100 μM in vitro, 50 mg/kg/body weight in vivo	Neuroprotection via activation of PI3k/Akt-ERK signalling, inhibition of apoptosis, and reduction of neuroinflammation	[177]
	Hypoxic-ischemic (H-1) Brain injury	Caspase-3; iNOS/COX-2	30 mg/kg	Neuroprotection via inhibition of apoptosis and neuroinflammation with reduced neuronal loss	[166]

## Data Availability

Not applicable.

## Abbreviations

AChE	Acetylcholinesterase Enzyme
AD	Alzheimer’s Disease
AKT	Protein Kinase B
ALS	Amyotrophic Lateral Sclerosis
AMPK	AMP-Activated Protein Kinase
AP-1	Activator Protein-1
ARE	Antioxidant Responsive Element
BACE-1	β-Secretase 1
Bax	Bcl-2-Associated Protein X
BBB	Blood Brain Barrier
BChE	Butyrylcholinesterase
BCL	B-Cell Lymphoma
BV2	Murine Microglial Cell Line
C/EBPβ	Ccaat Enhancer Binding Protein Beta
CAT	Catalase
cMyc	Cellular Myc Oncogene
CNS	Central Nervous System
CO	Carbon Monoxide
COX	Cyclooxygenase
COX-2	Cyclooxygenase-2
DL	Dalton’s Lymphoma
ELISA	Enzyme-Linked Immunosorbent Assay
ERK	Extracellular Signal-Regulated Kinase
FTH1	Ferritin Heavy Chain 1
GPX	Glutathione Peroxidase
GPX4	Glutathione Peroxidase 4
GSH	Glutathione
GSSG	Oxidized Gluthatione
GST	Glutathione S-Transferase
GSK-3β	Glycogen synthase kinase-3 beta
HD	Huntington’s Disease
HI	Hypoxic-Ischemic Brain Injury
HMGB-1	High Mobility Group Box 1
HO-1	Heme Oxygenase 1
IFN	Interferon
IKK	Inhibitor of Nuclear Factor-Κb (Iκb) Kinase
IL	Interleukin
iNOS	Inducible Nitric Oxide Synthase
JNK	Jun N-Terminal Kinase
Keap1	Kelch-Like ECH-Associated Protein 1
LDH	Lactate Dehydrogenase
LPO	Lipoperoxidation
MAPK	Mitogen-Activated Protein Kinase
MDA	Malondialdehyde
MMPs	Matrix Metalloproteinases
MND	Motor Neuron Diseases
MyD88	Myeloid Differentiation Primary Response Protein 88
NF-ĸB	Nuclear Factor-Kappa B
NMDA	N-Methyl D-Aspartate
NO	Nitric Oxide
NQO1	Quinone Oxidoreductase 1
Nrf2	Nuclear Factor Erythroid 2-Related Factor 2
PD	Parkinson’s Disease
PGE2	Prostaglandin E2
PI3K	Phosphatidylinositol 3-Kinase
PTGS2	Prostaglandin-endoperoxide synthase 2
PUFA	Polyunsaturated Fatty Acids
ROS	Reactive Oxygen Species
RSV	Respiratory Syncytial Virus
SAE	*Semecarpus anacardium* L., Extract
SCA	Spinocerebellar Ataxia
SCI	Spinal Cord Injury
SH-SY5Y	SK-N-SH Neuroblastoma Cell Line
SLC7A11	Solute Carrier Family 7-Member 11
SMA	Spinal Muscular Atrophy
SOD	Superoxide Dismutase
SP-1	Specificity Protein
SLC7A11	Solute Carrier Family 7 Member 11
TBI	Traumatic Brain Injury
TBK1	Tank-Binding-Kinase1
TFR1	Transferrin Receptor 1
TH	tyrosine hydroxylase
TIMP	Tissue Inhibitor of Metalloproteinase
TLR	Toll-Like Receptor
TNF	Tumor Necrosis Factor
t-SOD	Total Superoxide Dismutase
TTBK1	Tau-Tubulin Kinase 1
